# A service evaluation of short-term mentalisation based treatment for personality disorder

**DOI:** 10.1192/bjo.2021.974

**Published:** 2021-08-02

**Authors:** Niall M. McGowan, Nandana Syam, Debra McKenna, Steve Pearce, Kate E. A. Saunders

**Affiliations:** Department of Psychiatry, University of Oxford, Warneford Hospital, Oxford, UK; and Oxford Health NHS Foundation Trust, Warneford Hospital, Oxford, UK; Clinical Medical School, Medical Sciences Division, Academic Centre, John Radcliffe Hospital, University of Oxford, UK; Oxfordshire Complex Needs Service, Oxford Health NHS Foundation Trust, Oxford, UK; and Oxford Health NHS Foundation Trust, Warneford Hospital, Oxford, UK; Oxfordshire Complex Needs Service, Oxford Health NHS Foundation Trust, Oxford, UK; and Oxford Health NHS Foundation Trust, Warneford Hospital, Oxford, UK; Department of Psychiatry, University of Oxford, Warneford Hospital, Oxford, UK; and Oxford Health NHS Foundation Trust, Warneford Hospital, Oxford, UK; and NIHR Oxford Health Biomedical Research Centre, Oxford, UK

**Keywords:** Personality disorders, borderline personality disorder, group psychotherapy, mentalisation-based treatment, brief intervention

## Abstract

**Background:**

People with personality disorder experience long waiting times for access to psychological treatments, resulting from a limited availability of long-term psychotherapies and a paucity of evidence-based brief interventions. Mentalisation-based treatment (MBT) is an efficacious therapeutic modality for personality disorder, but little is known about its viability as a short-term treatment.

**Aims:**

We aimed to evaluate mental health, client satisfaction and psychological functioning outcomes before and after a 10-week group MBT programme as part of a stepped-care out-patient personality disorder service.

**Method:**

We examined routinely collected pre–post treatment outcomes from 176 individuals (73% female) aged 20–63 years, attending a dedicated out-patient personality disorder service, who completed MBT treatment. Participants completed assessments examining mentalising capacity, client satisfaction, emotional reactivity, psychiatric symptom distress and social functioning.

**Results:**

Post-MBT outcomes suggested increased mentalising capacity (mean difference 5.1, 95% CI 3.4–6.8, *P* < 0.001) and increased client satisfaction with care (mean difference 4.3, 95% CI 3.3–5.2, *P* < 0.001). Post-MBT emotional reactivity (mean difference −6.3, 95% CI −8.4 to −4.3, *P* < 0.001), psychiatric symptom distress (mean difference −5.2, 95% CI −6.8 to −3.7, *P* < 0.001) and impaired social functioning (mean difference −0.7, 95% CI −1.2 to −0.3, *P* = 0.002) were significantly lower than pre-treatment. Improved mentalising capacity predicted improvements in emotional reactivity (*β* = −0.56, *P* < 0.001) and social functioning (*β* = −0.35, *P* < 0.001).

**Conclusions:**

Short-term MBT as a low-intensity treatment for personality disorder was associated with positive pre–post treatment changes in social and psychological functioning. MBT as deployed in this out-patient service expands access to personality disorder treatment.

## Background

Access to treatment for personality disorders is a significant challenge in psychiatry. Personality disorder is common, with a prevalence of 4.4% in the general population^1^ and around 50% among psychiatric out-patients.^2^ Evidence-based treatments for personality disorder largely involve long-term psychological interventions and there are no medications specifically licensed for use in personality disorder, although the majority of individuals with the disorder are prescribed medication at some point.^3^ There are a number of efficacious psychotherapies for personality disorder,^4^ but service availability and treatment access are inconsistent. Only half of dedicated personality disorder services surveyed in England report equal patient access to services across localities where they are offered.^5^ The recommended intensity of treatments contributes to restricted access. In borderline personality disorder (BPD), the most commonly diagnosed personality disorder, psychotherapy lasting between 12 and 24 months is standard.^6^ Furthermore, national guidelines advise against brief psychological interventions (shorter than 3 months) because of expected negative reactions to treatment end or withdrawal in people with BPD.^7^ Thus, long waiting lists for treatment access and a paucity of available evidence-based short-term interventions results in substantial exclusion from needed treatment for people with personality disorder.

## Mentalisation-based treatment

The concept of mentalising refers to the ability to perceive and interpret behaviour as arising from mental states (e.g. emotions, thoughts, motivations) belonging to oneself and others.^8^ Mentalisation-Based Treatment (MBT) is a manualised therapy developed by Bateman and Fonagy,^9^ based on psychodynamic approaches and attachment theory suggesting that the ability to mentalise develops differently in people with personality disorder as a function of early insecure attachments. Hence, non-mentalising or ‘prementalising’ stances are recapitulated in adulthood, leading to difficulty for individuals with personality disorder to reflect upon their own mental states and to perceive the mental states of others. Challenges to mentalising effectively are most pronounced when encountering and managing emotional distress. MBT was originally developed for BPD, and its efficacy has been demonstrated by several randomised controlled trials (RCTs).^10,11^ Its use has also been expanded to other personality disorders.^12^ A low-intensity, 10-week MBT programme, delivered within a longer democratic therapeutic community (DTC) model of treatment, is the routine introductory psychotherapy provided in the local-specialist personality disorder service within the Oxford Health National Health Service (NHS) Foundation Trust. In the context of a stepped-care model of service,^13^ MBT is used to assess the suitability of extended or alternative higher-intensity treatment. Service evaluation of this programme through routinely collected service user reported outcomes permits the opportunity to assess the suitability of MBT as an initial step in short-term treatment for personality disorder.

## Aims

To perform a service evaluation of short-term MBT as it is used in an out-patient personality disorder service, using routinely collected service user outcomes. We examined self-reported pre–post MBT changes in mentalising capacity, client satisfaction, emotional reactivity, general psychiatric health and social functioning impairment.

## Method

### Study design, treatment context and participants

We conducted a service evaluation of short-term group MBT intervention as delivered via a dedicated out-patient service unit for personality disorders, the Oxfordshire Complex Needs Service (OCNS) of the Oxford Health NHS Foundation Trust. In the UK, personality disorder services are arranged in tiers, in which higher tiers correspond with increasing intensity of intervention as required for the greater severity of symptoms and functional impairment. Tier 1 and tier 2 consists of primary care and mainstream community mental health teams, respectively; tier 2 offers short- to medium-term psychosocial interventions. The OCNS is a tier 3, or local-specialist, service that offers dedicated community-based treatment for personality disorder in the counties of Oxfordshire and Buckinghamshire. Typically, service users are referred to the OCNS because they have more severe difficulties than those addressed in non-specialist service tiers, and may benefit from greater intensity intervention with a greater emphasis on therapeutic setting and supporting effective engagement. MBT is the introductory treatment modality initially provided to new service users, and is offered as part of a longer treatment trajectory within the service. The ability of service users to understand and implement MBT strategies to manage distress and achieve improvement of function in their day-to-day lives is used by team therapists to recommend next-step intervention groups appropriate to individuals' needs. This may involve an alternative therapeutic modality (cognitive analytic therapy) or a higher-intensity and longer duration treatment (emotional skills group and an 18-month DTC intervention), as judged appropriate by practitioners in the service. For this evaluation of MBT, we extracted participants’ pre-treatment (baseline) and post-treatment self-reported assessment outcomes that were collected as part of routine care.

Personality disorder diagnosis was confirmed at referral by OCNS practitioners using the SCID-II interview, and before admission to an MBT group. Participants were informed at admission and when the assessments were collected that clinical data may be used for service audit and evaluation purposes, and consented to the use of their de-identified data for this purpose. The service evaluation data described here did not involve any service user participation beyond their normal clinical management and involved no change to usual treatment within the service, and therefore did not require approval from a research ethics committee.

### Intervention

The MBT intervention consisted of ten structured group therapy sessions delivered over 11 weeks (one introductory session, ten MBT sessions). Groups consisted of 10–14 service users and at least two team therapists at each session. Sessions lasted for 2 h each week. MBT was delivered within a therapeutic community model of treatment, which has been described as ‘mentalisation-based therapeutic community’, and its format has been detailed previously.^14^ The content of MBT is based on the small-group component described in Bateman and Fonagy's original partial hospitalisation MBT trial, but was designed to be a standalone brief intervention, without parallel individual therapy. MBT sessions introduced the concept of mentalising to individuals (i.e. perceiving and interpreting the behaviour of oneself and others and their interpersonal relations as explained by intentional mental states) and focused on approaches to improve mentalisation, fostering strategies such as self-reflection, switching perspective and adopting an inquisitive stance. Further, an emphasis on the DTC values of communalism, democratisation, permissiveness and reality confrontation were incorporated into the small-group approach.^15^ Individual service users volunteered to act as chair for each session and emphasis was placed on encouraging group members to support and challenge each other's perspectives.

Weekly sessions were highly structured, and used exemplar scenarios to teach participants to recognise instances where mentalising capacity is challenged and to identify maladaptive non-mentalising modes (a,b,c) and unhelpful cognitive modes (d), as follows:

- Psychic equivalence mode: or ‘concrete mode’, where an individual interprets their thoughts and beliefs about reality as necessarily true such that no alternative perspectives are considered.
- Teleological stance: where an individual only considers the existence of a mental state where it is expressed in a physically or behaviourally evident manner. This leads to interpreting the actions of others as the only index of their mental state and ‘acting out’, in which individuals with personality disorder engage in risky or self-injurious behaviours to express their own mental state.
- Pretend mode: where an individual's mental state is disconnected from reality or becomes a substitute for reality. In this mode there is an overreliance on intellectualising/rationalising experiences, but individuals cannot integrate their cognitive appraisal of a mental state with reality or apply this understanding in a useful context.
- Hypermentalising: where an individual's ability to mentalise is adversely affected because they are fixated on or preoccupied with specific interpretations about possible motivations and mental states of themselves or others.

All OCNS practitioners delivering MBT were registered mental health professionals trained in the modality with extensive experience of working with people with personality disorder. Intervention progress and case-load were discussed at supervision meetings held fortnightly, supervised by an experienced consultant psychiatrist (S.P.).

### Assessments

Participants were asked to complete routine self-reported psychometric assessments before the introductory group MBT session. Assessments were completed either through an online patient-reported outcome platform (Patient Owned Database) or by pen and paper. Participants were asked to complete the assessments again after treatment ended (week 11). To allow for late paper responses, we only extracted information from baseline assessments returned up to week 3 and post-treatment assessments returned up to 9 weeks after treatment end (i.e. week 20).

The instruments used in assessment were as follows. The Mentalization Questionnaire (MZQ) is a 15-item questionnaire assessing an individual's ability to mentalise.^16^ Statements reflect instances of non-mentalising and are endorsed on a 0–4 Likert scale, with a total maximum score of 60. Items are reverse scored such that higher scores indicate better mentalising capacity. The Client Satisfaction Questionnaire (CSQ-8) is an eight-item questionnaire assessing satisfaction with care.^17^ Higher scores indicate greater client satisfaction. As baseline assessments were before treatment, participants were asked to rate their care satisfaction in relation to the service that they previously received. The Emotional Reactivity Scale (ERS) is a 21-item questionnaire assessing reactivity of emotions based on their intensity, sensitivity and persistence.^18^ Higher scores reflect greater emotional reactivity. The General Health Questionnaire (GHQ-12) is a 12-item questionnaire assessing non-specific psychiatric symptoms relating to impairment of function or novel or distressing symptoms (e.g. problems with concentration, sleep and psychosocial functioning).^19^ Items are scored with the Likert method (scored 0–3), with a maximum score of 36 and higher scores indicating worse outcome. The Social Functioning Questionnaire (SFQ) is an eight-item questionnaire assessing social functioning in a range of contexts (e.g. relationships, work and domestic tasks).^20^ The scale has a maximum score of 24, with greater scores indicating worse social functioning.

Additionally, participants completed the McLean Screening Instrument for Borderline Personality Disorder (MSI-BPD) before commencing treatment.^21^ The MSI-BPD is a ten-item inventory that screens participants for BPD symptoms. A score of seven or higher indicates a positive screen for BPD. As the instrument is a dichotomous checklist rather than a scale, it was not included as a treatment outcome.

### Data analysis

Analyses focused on assessing post-treatment changes of outcome measures compared with baseline, and exploring the degree to which outcome improvement was related to improvement in mentalising capacity. Paired-samples *t*-tests comparing baseline with post-treatment measures were used to assess improvement on outcome measures. We report the mean difference of outcome measure, Cohen's *d* effect size estimates and corresponding 95% confidence interval for both. Data distributions were approximately normal for all outcomes, but CSQ-8 and ERS scores showed modest negative skew at baseline and post-treatment assessment points, respectively. Thus, *t*-test results for these outcomes were confirmed with the non-parametric Wilcoxon signed-rank test. Hierarchical linear regression models were used to assess change in MZQ score as a predictor of outcome change. Demographic characteristics (gender and age) were inserted at step 1, with the addition of MZQ score change at step 2, with changes in CSQ-8, ERS, GHQ-12 and SFQ used as the dependent variable in each separate model. To control for family-wise error rate from assessing five separate outcome measures, we applied a Bonferroni correction to the alpha threshold (0.05/5) to determine values of *P* < 0.01 as statistically significant. All analyses were performed with SPSSversion 25 for Windows (IBM Corporation) or JASP version 0.11 for Windows (JASP Team, University of Amsterdam, The Netherlands; see https://jasp-stats.org/).

## Results

### Sample characteristics

Outcome data comprised responses from 176 service users (73% female) aged between 20 and 63 years, with a mean age of 37.9 (s.d. 11.1) years. The proportion of returned outcomes for each measure is presented in Table 1. The mean time at which post-treatment measures were recorded was 12.5 (s.d. 2.75) weeks after baseline assessments were returned. The proportion of assessments returned varied by questionnaire and was >60% for each measure. Consequently, we used pairwise exclusion for missing responses to minimise data loss. MSI-BPD assessment indicated that 82% of the sample screened positive for BPD, which was consistent with the previously reported high prevalence of BPD among patients attending the OCNS.^22^ After BPD, the other most common personality disorder diagnoses (including comorbid diagnoses) among individuals using the service were avoidant (67%), paranoid (36%), dependent (20%), narcissistic (17%) and obsessive–compulsive (16%).^22^

**Table 1:** Proportion of responses for questionnaire outcomes

Outcome measure response rate	*n* (%)
MZQ	138 (78)
CSQ-8	111 (63)
ERS	142 (81)
GHQ-12	120 (68)
SFQ	172 (97)

Questionnaire responses were available from 176 individuals that completed the mentalisation-based treatment intervention at the Oxfordshire Complex Needs Service. The number of returned questionnaires and proportion of the total number of participants is shown in the table above. MZQ, Mentalization Questionnaire; CSQ-8, Client Satisfaction Questionnaire; ERS, Emotional Reactivity Scale; GHQ-12, General Health Questionnaire; SFQ, Social Functioning Questionnaire.

### Treatment outcomes

Baseline and post-treatment self-reported assessment outcomes are presented in Table 2. The evaluation data suggest pre–post changes in participants’ mental health and psychological functioning following completion of short-term MBT intervention. Mentalisation capacity was higher (mean difference MZQ score 5.1, 95% CI 3.4–6.8, *P* < 0.001) at post-treatment compared with baseline, as was satisfaction with care (mean difference CSQ-8 score 4.3, 95% CI 3.3–5.2, *P* < 0.001). Lower levels of emotional reactivity (mean difference ERS score −6.3, 95% CI −8.4 to −4.3, *P* < 0.001), psychiatric symptom distress (mean difference GHQ-12 score −5.2, 95% CI −6.8 to −3.7, *P* < 0.001) and impaired social functioning (mean difference SFQ score −0.7, 95% CI −1.2 to −0.3, *P* = 0.002) were reported at post-treatment compared with baseline. Supplementary analyses confirmed that significant pre–post treatment effects were also detected by a non-parametric median rank comparison of CSQ-8 and ERS scores. To determine whether response bias arising from non-returned questionnaires could have influenced results, we conducted a series of supplementary analyses exploring whether missing CSQ-8, GHQ-12, MZQ and ERS status affected pre–post change on each outcome (Supplementary Tables 1–4 available at https://doi.org/10.1192/bjo.2021.974). Missing SFQ status was not examined because of a 97% return rate. Respondents who did not return a CSQ-8 response appeared to show greater improvement in ERS score (*P* = 0.002; Supplementary Table 1), and respondents who did not return an ERS response trended toward greater improvement in SFQ score (*P* = 0.024 at Bonferroni-uncorrected alpha; Supplementary Table 4).

**Table 2:** Baseline versus post-treatment outcome measures

Outcome	Baseline	Post-MBT	Mean difference [95% CI]; *P*-value	Effect size, Cohen's *d* [95% CI]
	Mean (s.d.)	Mean (s.d.)		
MZQ	21.8 (11.1)	26.9 (11.8)	5.1 [3.4–6.8]; *P* < 0.001	0.51 [0.33–0.69]
CSQ-8	20.7 (5.2)	24.9 (4.3)	4.3 [3.3–5.2]; *P* < 0.001	0.85 [0.64–1.07]
ERS	63.2 (13.7)	56.8 (17.1)	−6.3 [−8.4 to −4.3]; *P* < 0.001	0.50 [0.33–0.68]
GHQ-12	21.9 (7.6)	16.7 (8.2)	−5.2 [−6.8 to −3.7]; *P* < 0.001	0.63 [0.43–0.82]
SFQ	14.7 (3.7)	13.9 (4.1)	−0.7 [−1.2 to −0.3]; *P* = 0.002	0.24 [0.09–0.39]

MBT, mentalisation-based treatment; MZQ, Mentalization Questionnaire; CSQ-8, Client Satisfaction Questionnaire; ERS, Emotional Reactivity Scale; GHQ-12, General Health Questionnaire; SFQ, Social Functioning Questionnaire.

### Mentalisation as a predictor of pre–post symptom change

Hierarchical linear regression model summaries are presented in Table 3. Analyses revealed that pre–post intervention change in mentalising capacity was a significant predictor of improved emotional reactivity and social functioning.

**Table 3:** Hierarchical linear regressions predicting outcome measure improvement, using improvement in mentalising capacity

Outcome, *n*	Step	*R*^2^ adjusted	Δ*R^2^*	*F*	*P-*value	Predictor	*B*	s.e.	*β*	*P*-value
Dependent Variable: ΔCSQ-8 *n* = 86	1	0.018	0.006	0.258	0.774					
						Gender	−0.39	1.43	−0.03	0.784
						Age	−0.03	0.05	−0.08	0.496
	2	0.03	0.000	0.177	0.912					
						Gender	−0.39	1.14	−0.03	0.786
						Age	−0.03	0.05	−0.08	0.493
						ΔMZQ	0.01	0.06	0.02	0.88
Dependent Variable: ΔERS *n* = 138	1	0.031	0.045	3.184	0.045					
						Gender	5.97	4.21	0.21	0.013
						Age	0.05	0.09	0.04	0.636
	2	**0.348**	**0.317**	**25.329**	**<0.001**					
						Gender	**6.07**	**1.95**	**0.22**	**0.007**
						Age	0.06	0.08	0.06	0.431
						ΔMZQ	**−0.71**	**0.09**	**−0.56**	**<0.001**
Dependent Variable: ΔGHQ-12 *n* = 101	1	0.014	0.007	0.328	0.721					
						Gender	−1.18	1.81	−0.07	0.516
						Age	−0.05	0.08	−0.06	0.564
	2	0.024	0.000	0.222	0.881					
						Gender	−1.18	1.82	−0.07	0.647
						Age	−0.05	0.08	−0.06	0.568
						ΔMZQ	0.01	0.08	0.01	0.895
Dependent Variable: ΔSFQ *n* = 135	1	0.005	0.01	0.68	0.508					
						Gender	0.56	0.57	0.09	0.307
						Age	−0.01	0.02	−0.04	0.65
	2	**0.111**	**0.12**	**6.551**	**<0.001**					
						Gender	0.53	0.54	0.08	0.327
						Age	−0.01	0.02	−0.03	0.703
						ΔMZQ	**−0.1**	**0.02**	**−0.35**	**<0.001**

B indicates the unstandardised regression coefficient, with accompanying s.e.; *β* indicates the standardised regression coefficient; Δ represents the score-change (post-treatment – baseline). Gender is coded as zero for male and one for female. Bold indicates statistical significance at *P* < 0.01. CSQ-8, Client Satisfaction Questionnaire; MZQ, Mentalization Questionnaire; ERS, Emotional Reactivity Scale; GHQ-12, General Health Questionnaire; SFQ, Social Functioning Questionnaire.

For the model predicting change in ERS score, entry of MZQ change as a predictor at step 2 resulted in a total variance of 35% explained by the whole model, which was significantly greater than at step 1 (*R*^2^ change 0.317, *F*(1,134) = 66.53, *P* < 0.001). In the final adjusted model, gender (female) and MZQ score change were statistically significant predictors of change in ERS score (*β* = 0.22, *P* = 0.02 and *β* = −0.56, *P* < 0.001, respectively); part correlation indicated that 33% of the variance in emotional reactively change was uniquely explained by change in mentalising capacity.

**Fig. 1:**
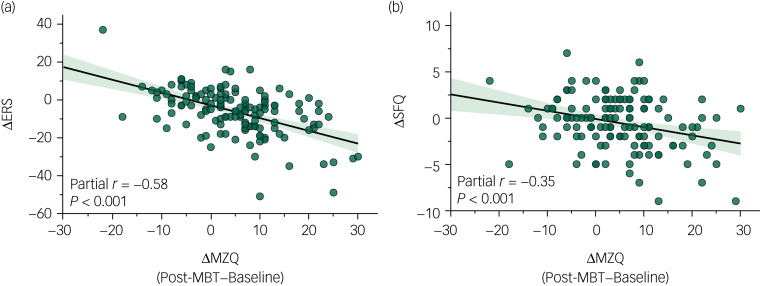
Association between mentalisation change and change in emotional reactivity and social functioning. Scatterplots show partial correlations between mentalisation change and (a) emotional reactivity and (b) social functioning, controlling for gender and age. Positive score-change on the MZQ horizontal axis indicates improved mentalising capacity. Negative score-change on outcome vertical axes indicates improvements in emotional reactivity (ERS) and social functioning (SFQ). ERS, Emotional Reactivity Scale; MBT, mentalisation-based treatment; MZQ, Mentalization Questionnaire; SFQ, Social Functioning Questionnaire.

For the model predicting change in SFQ score, entry of MZQ change as a predictor at step 2 resulted in a total variance of 11% explained by the whole model, which was significantly greater than at step 1 (*R*^2^ change 0.12, *F*(1,131) = 18.11, *P* < 0.001). MZQ score change was the only statistically significant predictor of change in SFQ score in the final model (*β* = −0.35, *P* < 0.001). Partial correlations between MZQ change and dependent variables controlling for age and gender are depicted in Fig. 1 (ERS: partial *r* = −0.58, *P* < 0.001; SFQ: partial *r* = −0.35, *P* < 0.001). Regression analyses assessing change on CSQ-8 and GHQ-12 outcomes did not show any meaningful association with mentalising capacity.

## Discussion

Short-term group therapy consisting of a 10-week MBT programme was associated with positive pre–post changes in mentalising capacity, emotion regulation, social functioning, psychiatric symptom distress and client satisfaction for people with personality disorder. Improved mentalising capacity predicted changes in emotional reactivity and social functioning.

### Intervention intensity

Few studies have examined brief interventions for personality disorder, presumably because of the chronic course of disorder, a complex symptomatology and guidelines that caution against low-intensity treatment. The present findings suggest that short-term MBT, delivered routinely as an initial introductory intervention within a stepped-care DTC model of service, is associated with positively directed self-reported symptom and functional change in people with personality disorder. Thus, in the context of the OCNS service evaluated here, short-term MBT appears to be a useful initial strategy for expanding treatment access for people with personality disorder before more intensive extended care.

There have been few service evaluation studies examining treatments for personality disorder. Previous work examining brief psychological treatments for personality disorder has focused on service-based outcomes such as reduced treatment cost and decreased hospital utilisation.^23,24^ Huxley et al recently presented service evaluation data showing that brief psychological treatment is also associated with improvements in clinical outcomes such as borderline symptoms, suicidal ideation and quality of life.^25^ The current study is the first to conduct a service evaluation on the use of short-term MBT applied as a routine part of care in the OCNS. There is a lack of similar service evaluation studies examining pre–post changes in mental health and psychosocial outcomes over time in people receiving short-term MBT intervention. Most work in this area has instead looked at longer duration treatment, often specifically for BPD.

Evidence-based studies examining MBT have done so for BPD, and involve long-term treatment durations (over 12 months).^10^ In-patient MBT studies as short as 4-weeks have reported beneficial outcomes from treatment,^26,27^ but findings from these studies cannot be extrapolated easily to out-patient settings because of the differences in intensity of care and use of a combination of other therapies, such as dialectical behaviour therapy (DBT). RCT studies of brief psychological interventions for personality disorder have been conducted primarily among people with BPD. A recent meta-analytic review of this literature also shows a predominance of short-form DBT or therapies that include substantial DBT-inspired components.^28^ However, no studies examining short-term MBT exclusively were identified.

Few RCTs exist examining the efficacy of other therapies applied as brief interventions for groups with BPD and mixed personality disorders. Korrelboom et al examined a 7-week adjunctive Competitive Memory Training (COMET) programme compared with treatment as usual in a group of mixed personality disorder diagnoses,^29^ Blum et al examined a 20-week adjunctive Systems Training for Emotional Predictability and Problem Solving (STEPPS) treatment compared with treatment as usual in BPD,^30^ and most recently, Crawford et al examined a six-to-ten session structured psychological support (SPS) programme compared with treatment as usual in a mixed cohort of people with personality disorder.^31^ SPS draws on techniques used in MBT and therefore, out of the RCT work highlighted above, might be considered the closest to the MBT intervention evaluated in the OCNS in terms of therapeutic focus and treatment duration. Innovative brief interventions investigated in the aforementioned RCT studies have demonstrated improvements in specific symptom outcomes, social functioning and patient well-being. Thus, the post-MBT changes that we describe in this service evaluation are clinically intuitive and correspond with trial data that examine the efficacy of treatment.

However, as this was a non-randomised and uncontrolled study, we cannot determine that improvement of outcomes pre–post intervention was as a result of treatment benefit *per se*. Replication with a matched control group is necessary to confirm an efficacy signal compared with waiting list or active control treatment. Meta-analysis of BPD studies suggests that treatment effect sizes are greater for brief interventions delivered adjunctively and smaller when compared withmanualised control interventions.^28^ Thus, treatment efficacy of intervention beyond that of structured contact is presently unclear, and is an additional consideration for future trials examining short-term MBT. The duration of the programme assessed here is also similar to that of the mean intervention length of brief intervention studies identified by review.^28^ Future clinical trials that compare short-term with longer-term MBT are required to clarify the optimal length of brief interventions in personality disorder. Juul et al proposed an RCT to examine this point in BPD.^32^ Future work targeting a mixed cohort of people with personality disorder is also required to confirm the present findings suggesting symptom improvement at post-intervention.

### Treatment outcomes

Improved symptom outcomes reflected domains of emotional dysregulation (emotional reactivity), interpersonal dysfunction (social functioning) and mixed psychiatric symptom distress (e.g. depressed mood, disturbed sleep and difficulty concentrating). Our results agree with findings indicating psychological treatment benefits for personality disorder. Psychotherapy reduces mood instability,^11^ and several studies demonstrate that MBT associates specifically with improved social functioning.^10,11^

Pre- to post-treatment effect sizes in this study were predominantly of medium strength, and small for social functioning. Similarly designed cohort studies (i.e. those using within-group comparisons) for MBT-based interventions have reported larger treatment effects;^33,34^ however, this may be because treatment duration was also substantially longer. In the context of a whole service model of stepped-care, MBT, as delivered here, is not intended to be a complete course of treatment. Further treatment gains are likely achievable with extended care.

A frequent limitation of studies examining MBT is that parallel measurement of mentalising change is not generally assessed. Consequently, little is known about the actual therapeutic mechanisms of treatment.^10^ Assessment of mentalising improvement was an explicit outcome in evaluating this service.

Expectedly, mentalising capacity improved from baseline assessment to post-MBT assessment. Furthermore, improvements in mentalising capacity predicted improvements in emotional reactivity and social functioning. That mentalising was associated with improvements on these domains is consistent with the conceptual basis for intervention. Mentalising involves developing reflective insight into one's own mood state, and forms an important part of social cognition in the context of perceiving and understanding the motivations and perspectives of others.

It is hypothesised that social and cognitive difficulties moderated by reduced mentalisation underpin the severe emotional dysregulation and interpersonal dysfunction that are core symptoms of BPD. MBT has also been suggested as a viable treatment approach for other Cluster B personality disorders, where disturbances are characterised primarily by emotional dysregulation.^12,35^ Concordant with our service user outcomes, mentalising improvements are associated with reduced interpersonal distress.^36^ Emotional dysregulation and interpersonal dysfunction are primary reasons for people with personality disorder to seek treatment. Consequently, establishing which therapies are effective at targeting these symptom domains in future RCT studies is important for developing appropriate short-term treatments where service users derive maximum benefit.

It is unclear why we did not detect an association between mentalising capacity and general psychiatric symptom distress captured by the GHQ-12, despite significant improvement on this outcome. The diverse and non-specific symptom characteristics captured by the instrument may lack a clear symptom adjacency to mentalisation to show a proportionate association. Improvement on this outcome may not arise from mentalising capacity *per se*, but because of improved psychosocial functioning following improvement on proximal domains such as emotional reactivity and social functioning, as described above. Indeed, previous studies involving people with personality disorder have shown close associations between mood instability and other psychiatric symptoms, such as sleep disturbance^37^ and impaired neurocognitive performance.^38^ Improvements on this measure may have been also moderated by the DTC components of the intervention: communal experience and democratisation of group member roles are plausible contributors to improvement in self-reported psychosocial functioning (reflected partially through GHQ-12 and the aforementioned SFQ scores).

Satisfaction with care improved from baseline to post-treatment assessment, and showed the largest improvement of all outcomes assessed. Poor experience of care in mental health settings is an important factor influencing engagement with treatment and premature withdrawal from care.^39^ Furthermore, this can be additionally challenging for personality disorder services because people with personality disorder tend to express lower satisfaction with care, possibly resulting from unfulfilled expectations about symptom improvement.^40^ In the context of the service evaluated, satisfaction with short-term intervention is an important outcome that may have implications for service user engagement and retention at OCNS. We highlight that within a whole model of treatment for personality disorder, positive introductory experience with psychotherapy is likely to play an important role in maintaining engagement with follow-on extended care. As above, the democratised and communal nature of therapy delivered via a DTC model may account for improvement rather than mentalising improvement, which did not predict client satisfaction. Shared sense of endeavour among service users as well as group facilitation by practitioners that were experienced personality disorder clinicians may also have affected client satisfaction with care. However, without empirical confirmation of the above in this service, these are presently only speculative possibilities. Future qualitative methods may aid the identification of which group member and therapist experiences contribute to pre–post self-reported change.

### Strengths and limitations

To our knowledge, this is the first service evaluation study examining short-term MBT for personality disorders. Few service evaluation studies examine pre–post intervention changes in mental health and psychosocial functioning. In the context of an introductory psychotherapy programme offered to service users at the OCNS, short-term MBT appears to achieve favourable self-reported outcomes compared with when people are assessed at the beginning of treatment. Thus, short-term MBT offered through the OCNS might be a helpful first-step treatment for people with personality disorder who are referred to the service. A strength of this evaluation was the inclusion of the MZQ measure of mentalising capacity; seldom have empirical studies (including RCTs) used a parallel measure of mentalising function. That mentalising capacity was improved, and that improvement was associated with positive change on other outcomes further suggests that short-term MBT fulfils its purpose as an initial treatment targeting mentalising difficulties for people attending the OCNS.

This work has several limitations. First, we lack the experimental control necessary to evaluate the efficacy of short-term MBT in a placebo-controlled manner. Thus, we cannot infer that the pre–post improvements in mental health and psychological functioning are necessarily because of the intervention or the individual therapeutic components of MBT, and could reflect regression to the mean. Treatment fidelity was also not assessed by external observation, as might be done in an RCT study. Furthermore, as a service evaluation, the purpose of this study was to examine the intervention in the context of the service in which it was provided, and therefore data collection was not intended to produce findings generalisable beyond the OCNS service. Second, as data collection was naturalistic, it also lacked the systematic approach that would be present in a cohort study with a clearly pre-defined design and *a priori* hypotheses. Data were available from people who completed the intervention and who returned self-reported assessments only, and therefore we were unable to assess loss to follow-up. Third, because of partial responses on self-reported outcome measures, we cannot exclude the possibility of response bias among participants. However, supplementary analyses determined that only CSQ-8 non-respondents showed any significant differences to questionnaire respondents, and this was unlikely to indicate positive response bias as CSQ-8 non-responders showed greater improvement. Future RCTs with an intention-to-treat design are needed to examine the efficacy of short-term out-patient MBT for personality disorder. As suggested recently by a contemporary meta-analysis of the BPD brief intervention literature,^28^ demonstrating treatment superiority of intervention above regular structured contact between clients and the service is also needed to corroborate the promising findings described here. Finally, the outcome measures collected by the service did not probe individual symptoms of personality disorder because of the diverse representation of diagnoses in the service that involve heterogenous core symptoms. Future studies incorporating measures of core symptom severity are required to disentangle whether complex individual symptoms (e.g. fears of abandonment, suicidality, impulsivity) are more or less sensitive to treatment, and to determine core symptom severity at the start of treatment.

In conclusion, we found that a short-term (10-week) out-patient MBT treatment programme for personality disorder was associated with significant pre–post changes in self-reported outcomes reflecting mental health, psychological functioning and satisfaction with care among service users. The short-term MBT intervention deployed at the OCNS may increase the availability of appropriate care for personality disorder and improve treatment access where, traditionally, long waiting lists result in delayed treatment and substantial exclusion from care. In this service, brief intervention is not considered a substitute for long-term treatment of personality disorder, but rather may be used within a stepped-care model of service to prioritise access to suitable psychological therapies and introduce service users to intervention.

## Data Availability

The data that support the findings of this study are available from the corresponding author, N.M.M., upon reasonable request.

## Supplementary material

For supplementary material accompanying this paper visit http://dx.doi.org/10.1192/bjo.2021.974. click here to view supplementary material
