# Supplementary material for: A service evaluation of short-term mentalisation based treatment for personality disorder
Source: BJPsych Open. 2021 Aug 2;7(5):e140. doi: 10.1192/bjo.2021.974 (PMC8358973; doi:10.1192/bjo.2021.974)
Supplement: Supplementary file 1 [file S2056472421009741sup001.pdf]

SUPPLEMENTARY MATERIAL

A service evaluation of short-term mentalization based treatment for personality disorder

Niall M McGowan*[1,2], Nandana Syam[3], Debra McKenna[2], Steve Pearce[2], and Kate E A Saunders[1,2,4]

1. Department of Psychiatry, University of Oxford, Warneford Hospital, Oxford, UK
2. Oxford Health NHS Foundation Trust, Warneford Hospital, Oxford, UK
3. Oxford University Clinical School, Academic Centre, John Radcliffe Hospital, Oxford, UK
4. NIHR Oxford Health Biomedical Research Centre, Oxford, UK

ORCID IDs

Niall M McGowan (0000-0001-8183-8558)

Steve Pearce (0000-0002-6975-085X)

Kate EA Saunders (0000-0003-3448-9927)

*Corresponding author: Niall M McGowan

Address: Department of Psychiatry, University of Oxford, Warneford Hospital, Oxford, OX3 7JX, United Kingdom

Email: niall.mcgowan@psych.ox.ac.uk

Phone: +44 1865 613196

Wilcoxon signed-rank test analysis

To examine the robustness of the significant effects detected for CSQ-8 and ERS outcomes using paired-sample t-tests, we conducted follow-up Wilcoxon signed-rank tests which were robust to distribution skew detected at the post-MBT assessment point. The median post-intervention CSQ-8 rank (Mdn = 25) was significantly higher compared to the baseline rank (Mdn = 22), indicating a significant increase in client satisfaction post-MBT (z = -7.34, P<0.001). The median post-intervention ERS rank (Mdn = 60.5) was significantly lower than at baseline (Mdn = 66), indicating a significant decrease in emotional reactivity at the post-MBT assessment point (z = -5.62, P<0.001).

Assessing response bias by non-returned questionnaires

To examine the possibility of response bias among the cohort we conducted a comparison of questionnaire respondents versus non-respondents based on the availability of CSQ-8 score (Supplementary Table 1), GHQ-12 score (Supplementary Table 2), MZQ score (Supplementary Table 3), and ERS score (Supplementary Table 4).

Supplementary Table 1. CSQ-8 respondents versus non-respondents

	Response	No response				
**Outcome**	**Mean (SD)**	**Mean (SD)**	***t***	***df***	**Mean difference**	***P***
ΔMZQ	4.5 (10.1)	6.1 (9.9)	0.88	136	1.55	0.379
ΔERS	-3.9 (12.0)	-10.6 (12.6)	-3.12	140	-6.65	0.002
ΔGHQ-12	-4.0 (8.8)	-6.6 (7.6)	-1.68	118	-2.55	0.097
ΔSFQ	-0.6 (3.0)	-1.0 (3.1)	-0.84	170	-0.40	0.405

Supplementary Table 2. GHQ-12 respondents versus non-respondents

	Response	No response				
**Outcome**	**Mean (SD)**	**Mean (SD)**	***t***	***df***	**Mean difference**	***P***
ΔMZQ	5.2 (10.6)	4.8 (8.8)	-0.19	136	-0.37	0.848
ΔCSQ-8	4.6 (5.4)	3.9 (4.5)	-0.68	109	-0.66	0.499
ΔERS	-7.6 (13.7)	-3.2 (8.8)	1.88	140	4.38	0.063
ΔSFQ	-0.9 (3.1)	-0.3 (3.0)	1.25	170	0.63	0.213

Supplementary Table 3. MZQ respondents versus non-respondents

	Response	No response				
**Outcome**	**Mean (SD)**	**Mean (SD)**	***t***	***df***	**Mean difference**	***P***
ΔCSQ-8	4.2 (5.1)	4.6 (4.8)	0.40	109	0.45	0.693
ΔERS	-6.5 (12.7)	-2.3 (9.6)	0.66	140	4.21	0.512
ΔGHQ-12	-5.6 (8.5)	-3.4 (7.5)	1.01	118	2.23	0.290
ΔSFQ	-0.6 (3.0)	-1.2 (3.4)	-1.01	170	-0.60	0.290

Supplementary Table 4. ERS respondents versus non-respondents

	Response	No response				
**Outcome**	**Mean (SD)**	**Mean (SD)**	***t***	***df***	**Mean difference**	***P***
ΔCSQ-8	4.0 (5.1)	5.4 (4.6)	1.11	109	1.35	0.270
ΔGHQ-12	-5.6 (8.5)	-3.1 (7.7)	1.17	118	2.51	0.244
ΔSFQ	-0.49 (3.0)	-1.8 (3.0)	-2.28	170	-1.33	0.024

*no cases existed where MZQ was returned but not ERS
